# Effects of time-of-day resistance training on muscle strength, hormonal adaptations, and sleep quality during Ramadan fasting

**DOI:** 10.3389/fnut.2024.1439738

**Published:** 2024-11-13

**Authors:** Raoua Triki, Abderraouf Ben Abderrahman, Iyed Salhi, Fatma Rhibi, Ayoub Saeidi, Abdullah Almaqhawi, Anthony C. Hackney, Ismail Laher, Urs Granacher, Hassane Zouhal

**Affiliations:** ^1^Higher Institute of Sport and Physical Education of Ksar-Said, University of Manouba, Tunis, Tunisia; ^2^Tunisian Research Laboratory “Sports Performance Optimization”, National Center of Medicine and Science in Sports (CNMSS), Tunis, Tunisia; ^3^Movement, Sport, Health and Sciences Laboratory (M2S), UFR APS, University of Rennes 2-ENS Cachan, Rennes, France; ^4^Department of Physical Education and Sport Sciences, Faculty of Humanities and Social Sciences, University of Kurdistan, Sanandaj, Kurdistan, Iran; ^5^Department of Family and Community Medicine, College of Medicine, King Faisal University, Al Hofuf, Saudi Arabia; ^6^Department of Exercise and Sport Science, University of North Carolina, Chapel Hill, Chapel Hill, NC, United States; ^7^Department of Anesthesiology, Pharmacology, and Therapeutics, Faculty of Medicine, University of British Columbia, Vancouver, BC, Canada; ^8^Department of Sport and Sport Science, Exercise and Human Movement Science, University of Freiburg, Freiburg, Germany; ^9^Institut International des Sciences du Sport (2I2S), Irodouer, France

**Keywords:** fasting, muscle performance, training, hormonal adaptation, sleep quality

## Abstract

**Objectives:**

We investigated the timing of resistance training (RT) during Ramadan fasting (RF) on muscle strength, hormonal adaptations, and sleep quality.

**Methods:**

Forty healthy and physically active male Muslims (age = 25.7 ± 5.6 years, body mass = 85.1 ± 17.5 kg, height = 175 ± 9 cm, BMI = 28.3 ± 5.7 kg/m^2^) were enrolled in this study and 37 completed pre and post-tests. Subjects were randomly allocated into two experimental groups. Group 1 (FAST, *n* = 20) completed an 8-week whole-body RT in the late afternoon (between 16 h and 18 h) while fasting. Group 2 (FED, *n* = 20) completed the similar RT protocol compared with FAST at night (between 20 h and 22 h). The following parameters were analyzed at various time-points: 2 weeks before the start of RF (T0), on the 15th day of Ramadan (T1), on the 29th day of Ramadan (T2), and 21 days after the last day of RF (T3) where both groups were in a fed state. One-repetition maximum tests (1-RM) were conducted for the squats (1-RM_SQ_), the deadlift (1-RM_DL_) and the bench press (1-RM_BP_). Sleep quality was assessed using the full Pittsburgh Sleep Quality Index (PSQI). Blood samples were taken to determine cortisol, testosterone and IGF-1 levels. Additionally, acute hormonal responses were evaluated before (BF), immediately after (AF), and 30 min after a RT session (AF-30 min) at T0, T1, T2, and T3.

**Results:**

Significant group-by-time interactions were identified for 1-RM_SQ_ (*p* = 0.001; effect size [ES] = 0.43) and 1-RM_DL_ (*p* = 0.001; ES = 0.36). *Post-hoc* tests indicated significant 1-RM_SQ_ (*p* = 0.03; ES = 0.12) and 1-RM_DL_ (*p* = 0.04; ES = 0.21) improvements from T0-T2 for FED. Additionally, significant group-by-time interactions were observed for the chronic effects on cortisol (*p* = 0.03; ES = 0.27) and testosterone levels (*p* = 0.01; ES = 0.32). *Post-hoc* tests indicated significant increases of cortisol levels among FAST at T1 and T2 compared to T0 (*p* = 0.05; ES = 0.41, *p* = 0.03; ES =  0.34) and a significant increase in cortisol levels in FED at T1 (*p* = 0.05; ES = 0.29) and T2 (*p* = 0.04; ES = 0.25). However, the observed increase was lower compared to FAST. *Post-hoc* tests also indicated significant increases of testosterone only among FED at T2 (*p* = 0.04; ES = 0.31). A significant group-by-time interaction was found for the acute effect of exercise on cortisol level (*p* = 0.04; ES = 0.34). The cortisol level immediately after RT was higher in FAST only at T1 (*p* = 0.03; ES = 0.39) and T2 (*p* = 0.05; ES = 0.22) compared with T0. No significant group-by-time interactions were identified for sleep quality (*p* = 0.07; ES = 0.43).

**Conclusion:**

Muslims can safely practice RT during RF. However, training in a fed state during Ramadan might be more effective than during fasted state for the enhancement of maximal strength with better hormonal responses observed.

## Introduction

More than 1.9 billion healthy Muslims worldwide fast intermittently during the Ramadan month (RF) as one of the fundamental obligations of the Muslim faith according to the Pew Research Center (1). During this religious month, Muslims abstain from any type of food and fluid intake, engaging in sexual activities, and smoking from dawn *(suhoor)* to sunset *(iftar)* (2, 3). Thus, consuming food and drinks during RF is restricted to the hours of darkness (4). Additionally, the timing of meals and caloric restriction have previously been associated with changes in physiological, chronobiological, and social behaviors (5–8). Hormonal secretions (catecholamines, steroids, growth, and gut hormones) and sleep–wakefulness patterns are modified by circadian changes in food intake and social habits during RF (3, 9, 10).

The effect of engaging in sports during RF was underlined during the London Olympic Games 2012 and also the FIFA Soccer World Cup 2014, as they staged during the RF (11, 12). Some studies reported that training during RF is an effective strategy to improve physical fitness and overall health despite the dietary challenges (13, 14). Regular physical activity during fasting can improve cardiovascular health, reduce the risk of chronic diseases, and improve insulin sensitivity (15). Additionally, integrating sports and physical activity into the life style routine during RF helps to increase spiritual awareness and mindfulness. Many Muslims find that training during RF helpful to connect with their bodies and their faith (16).

Some researchers observed that low energy intake alters the availability and utilization of energetic substrates that can have adverse effects on metabolic, immune, and inflammatory responses and may thus lead to impaired physical fitness (17–20). In addition, insufficient sleep can affect physical fitness by increasing fatigue perception and decreasing mental alertness in response to the same exercise load during RF (8, 21).

It is therefore important to implement effective strategies such as a balanced diet during the non-fasting period, scheduling sports activities, and exercise outside fasting hours, and reducing the intensity and duration of exercise in order to maintain physical performance and adapt to the physiological changes associated with fasting (22).

The maintenance of physical exercise during RF is also challenging for athletes and physically active people who continue resistance training (RT). While there is a plethora of research on the effects of RT applied during RF on measures of body composition and muscular fitness such as muscle power, strength, and local muscular endurance (23, 24), there is limited data on the effects of RT during RF on sleep patterns and hormonal adaptations. Accordingly, we examined the timing of RT during RF when RT is performed either after breaking fast during a fed state to minimize fasting-induced performance degradation (25, 26), or during a fasting state to prevent sleep and hormonal perturbation after night training that could negatively affect muscle strength (27, 28). Additionally, researchers from previous studies also investigated the acute effects of exercise during fasting on hormonal secretion of growth hormones (GH), testosterone, and cortisol levels, which are higher after exercise and remain elevated 60 min after exercise during caloric restriction (29, 30).

Therefore, it is important to choose the right time of day for RT training during RF to minimize the effects of changes in mealtimes and the disruption of hormonal responses and sleep quality during this month, thus offering better physical performance and muscle maintenance. This study aimed to investigate the effects of performing RT in fasted state (FAST, i.e., training before breaking fast) or fed state (FED, i.e., training after breaking fast) on measures of muscle strength, sleep quality, and chronic hormonal responses in healthy and physically active young male adults. A secondary aim was to verify the effects of RT during FED or FAST on acute hormonal responses. With reference to the literature (23, 24), it was hypothesized that RT could be safely practiced during RF with both schedules; and that training after breaking fast may increase muscle strength while preserving normal hormonal responses.

## Methods

### Experimental approach to the problem

This longitudinal study was conducted during the Ramadan of the lunar year 1442 *Hijri* (from March 12th to April 13th, 2021), with 15 ± 1 h of fasting every day. The study was conducted in Tunisia, where daytime temperatures during the study period were ~ 23 ± 4°C with a relative humidity of 65 ± 5%.

**Figure 1:**
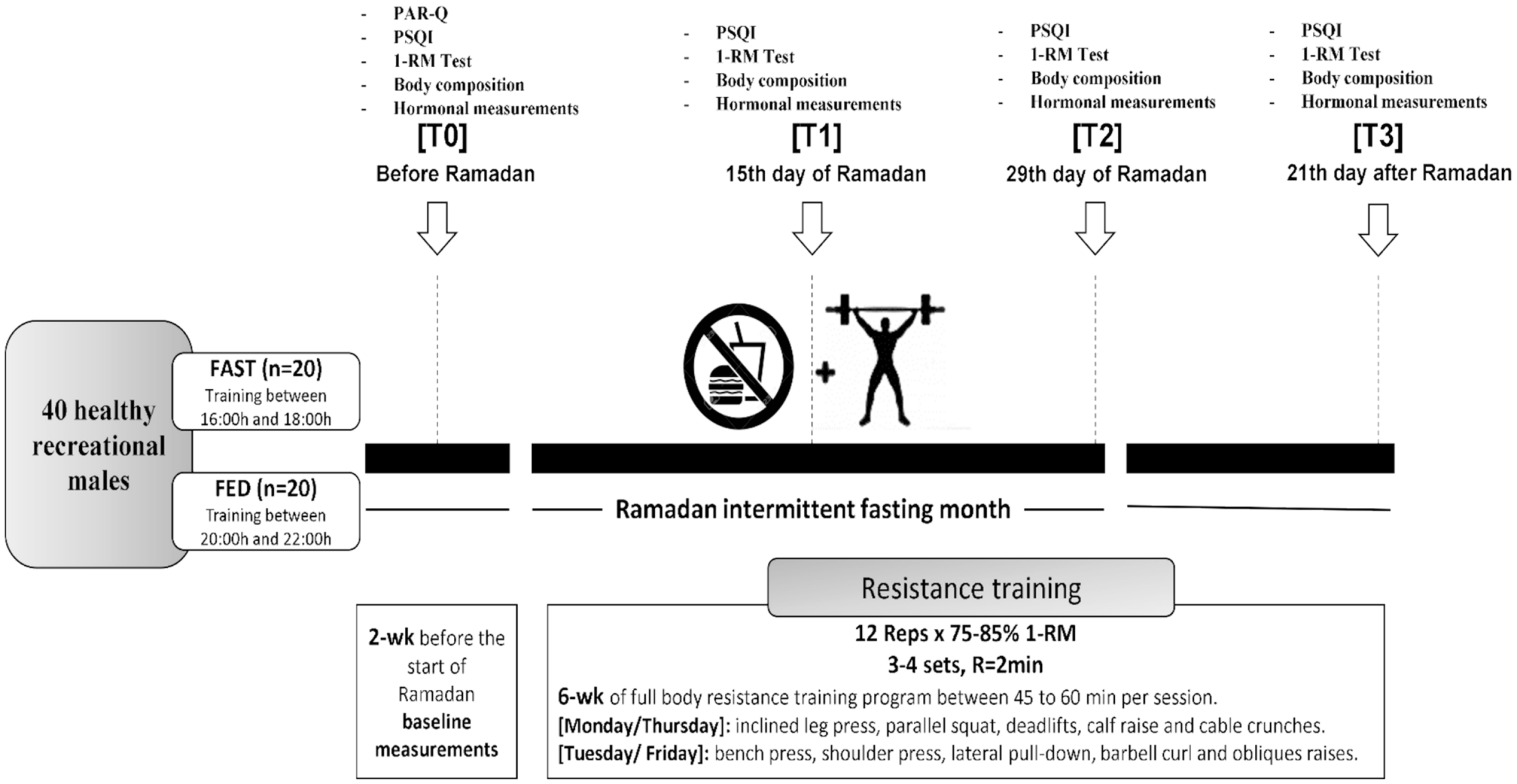
Study design. PSQI, Pittsburgh Sleep Quality Index; PAR-Q, physical activity readiness questionnaire; 1-RM, one repetition maximum; R, rest; wk, week; reps, repetitions.

Data on maximal strength, sleep quality, and hormonal adaptations (cortisol, testosterone, and IGF-1) were collected at four different time points: 2 weeks before RF (T0), on the 15th day of (T1), on the 29th day of (T2), and 21 days after the end of Ramadan (T3). All tests were conducted in the same order and time of day during pre and post-tests (see Figure 1).

### Participants

**Figure 2:**
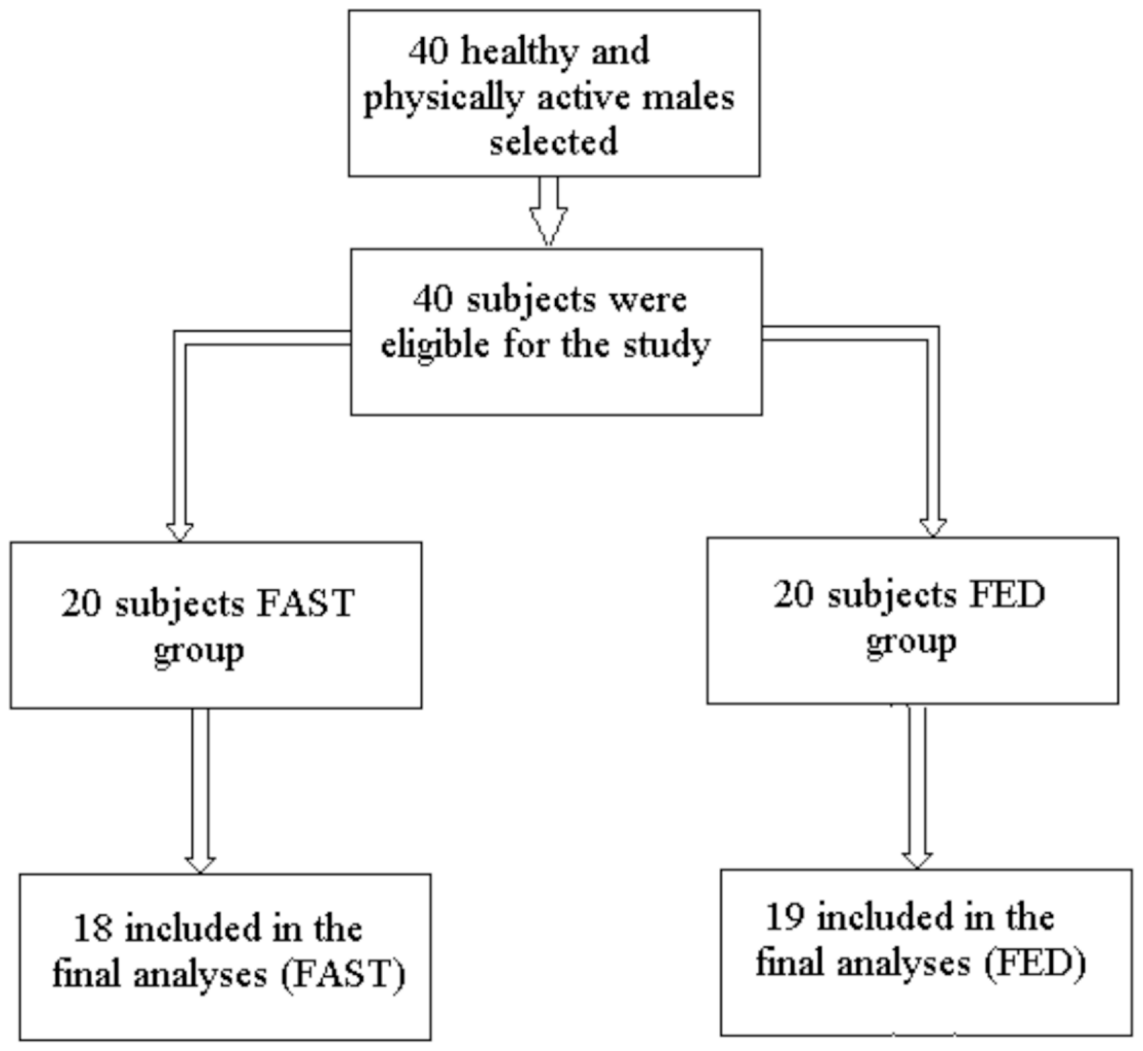
Flowchart of the sampling process.

The sample size of our study was determined using the power analysis program G*Power (version 3.1.9.3, University of Kiel, Kiel, Germany). The *a priori* power analysis was calculated using the F-test family (i.e., ANOVA repeated measures within-between interaction; power = 0.8, alpha = 0.05, effect size Cohen’s *f* = 0.3), and a related study that examined the effects of RF intermittent fasting on 1-RM performance (31). Outcomes from the power analysis indicated that a total sample size of 24 subjects to be sufficient (31). The sample size was increased to 40 to allow for potential dropouts of study participants (Figure 2).

Forty healthy and physically active males aged 25.7 ± 5.6 years (body mass = 85.1 ± 17.5 kg, height = 175 ± 9 cm, BMI = 28.3 ± 5.7 kg/m^2^) volunteered to participate in this study. Subjects were randomly assigned into two groups: FAST (*n* = 20) practiced RT in the afternoon before breaking fast (4–6 pm); FED (*n* = 20) practiced RT in the evening 1–2 h after breaking fast (8–10 pm) during RF. We added a control period of 21 days after the end of RF. During this time, both groups continued with the same training program at the same time of day under a normal nutritional regime (fed state). The study goal was to investigate whether the time-of-day training has an effect under the same nutritional conditions in either of the study groups.

Subjects were eligible for the study if they were healthy males aged between 18 and 35 years old, had at least 2 years of RT experience, lacked a history of cardiometabolic diseases or muscle injuries that could have precluded participation in the RT program, did not use medications, ergogenic aids, anabolic steroids or dietary supplements. Individuals had to refrain from other physical exercise during the study duration. All subjects had a medical examination and were fully informed about all experimental procedures, possible discomforts, risks, and benefits of the participation. Thereafter, they were kindly asked to sign the written informed consent form prior to the start of the study. The applied study procedures were in accordance with the principles outlined in the latest version of the Declaration of Helsinki. The study was approved by the local University Ethics Committee on Human Research of the University of Manouba, Tunisia (Ethics Code: Tn, UM2019-73).

Subjects visited the laboratory to become acquainted with all procedures before the start of data collection. The first day of testing was devoted to signing the informed consent form, answering the physical activity readiness questionnaire (PAR-Q), and the Pittsburgh Sleep Quality Index (PSQI), undergoing anthropometric measurements, and completing the one repetition maximum (1-RM) test. On the second test day, the 1-RM tests were repeated to determine the loads that were used during the RT programs and to assess test–retest reliability of the applied tests. During the third test day, blood samples were collected to analyze testosterone, cortisol, and the insulin-like growth factor-1 (IGF-1). The test sessions were separated by 48 h.

### Exercise program

A whole-body RT program was performed on 4 days per week (Monday, Tuesday, Thursday, and Friday) for 8 weeks (1 week before Ramadan, 4 weeks during Ramadan, and 3 weeks after the end of Ramadan). Each workout included five exercises that were repeated twice. The applied exercises on Monday and Thursday involved the inclined leg press, the parallel squat exercise, deadlifts, calf raises, and cable crunches. On Tuesday and Friday, subjects performed the bench press, the shoulder press, the lateral pull-down, the barbell curl, and obliques raises.

Each exercise session lasted 45–60 min and started with a general warm-up (10 min) and included a cool-down period (5–10 min) of low-intensity aerobic and dynamic stretching exercises. Subjects performed 4 sets × 12 repetitions at 75–85% of the 1-RM for every exercise with 2 min of passive recovery between sets and exercises. Exercise intensities were gradually increased during the 8-week training period, from 75% of the 1-RM at the start of the program to 85% of the 1-RM at week 3, until the end of the intervention. Each Subject was individually supervised by an experienced strength and conditioning specialist (RT) who managed load progression to ensure that each exercise was performed with adequate exercise technique during the concentric and eccentric phases of movement. Time under tension amounted to 2 s during the concentric phase and 2 s during the eccentric phase. The person responsible for exercise supervision monitored compliance and individual workout data for each exercise session (e.g., number of repetitions and sets, rest, movement velocity, and intensity).

### Anthropometric tests

Body composition was measured in the morning (10:00 a.m.) at T0, T1, T2, and T3 following an overnight fast (10:00 h) and after more than 36 h following the last exercise bout.

Height was measured using a stadiometer. Body mass was assessed using an electronic scale with a resolution of 50 g (Medisana®, Neuss, Germany). The percentage of body fat was determined with a Harpenden caliper (Baty international. RH15 9LR. England) in four skinfolds (biceps, triceps, subscapular, and suprailiac) (32). Lean body mass (LBM) was calculated as body mass minus body fat mass. All measurements were performed by the same investigator following standardized procedures (33).

### Assessment of sleep quality

Sleep quality was assessed directly after taking the anthropometric and body composition measurements at T0, T1, T2, and T3 using the full Pittsburgh Sleep Quality Index (PSQI) validated for the Arabic language (34). The global PSQI score is composed of seven components: subjective sleep quality (individual’s sleep quality perception), sleep latency (time between lying in bed and starting sleeping), sleep duration (period sleeping reported by the individual), habitual sleep efficiency (ratio between the time sleeping and the time in bed), sleep disturbance (problems to sleep expressed by subjects), sleeping medication (use of medication to sleep), and daytime dysfunction (intensity of diurnal somnolence) (35). The seven domains were separately scored from 0 (no difficulty) to 3 (very difficult), with a total or global score ranging from 0 to 21 to reflect sleep quality and disturbances over a 1-month period. Better sleep quality is indicated through have a lower global PSQI score, while higher scores indicate poor sleep quality.

### Maximal strength tests

The 1-RM tests for the squat, bench press, and deadlift exercises were determined for each subject at T0, T1, T2, and T3 to evaluate the development of maximal strength during the study. Additionally, 1-RM tests were re-assessed every 2 weeks to maintain the targeted exercise intensity (75–85% 1-RM). The 1-RM testing and the start of the training sessions were separated by 10 min of rest to allow for an appropriate recovery.

Subjects were asked to perform 10 repetitions with the estimated 10-RM as a warm-up before the start of the test for each exercise using previously described procedures (36, 37). The 1-RM was determined with a maximum of six attempts, with a 5-min rest period between each attempt (37). In addition, a 3-min rest was allowed between the tests. Pilot data were obtained on 2 test days from 40 subjects to determine test–retest reliability. Our data revealed the following ICCs 1-RM parallel squat (0.929; 95%CI lower 0.929–upper 0.996), 1-RM bench press (0.779; 95%CI lower 0.581–upper 0.883), 1-RM deadlift (0.886; 95%CI lower 0.783–upper 0.940).

### Blood samples

Four venous blood samples were taken at T0–T3. A heparinized catheter (Insyte-W, 1.1 mmo.d. × 30 mm, Biopol, Tunis, Tunisia) was inserted into an antecubital vein by using a 20-gauge needle and Vacutainer tubes before, at 0 min (immediately after), and 30 min after the training sessions. The blood was collected in test tubes containing EDTA and centrifuged at 1,500 × *g* for 10 min at 4°C, resultant serum was then removed and stored at −80°C until subsequently testosterone (nmol/L), cortisol (nmol/L), and IGF-1(ng/mL) were analyzed using radioimmunoassay commercial kits (Beckman Coulter and Diagnostic Systems Laboratories, France) according to the manufacturer’s procedures. The limits of sensitivity were 0.5 nmol/L for testosterone, 5.79 nmol/L for cortisol, and 3 ng/mL for IGF-1. The intra-assay coefficients of variation were between 0.9 and 4.6% for testosterone, between 2.24 and 6.01% for cortisol, and between 6.3 and 6.8% for IGF-1. All samples were assayed in duplicate and were decoded only after the analyses were completed (i.e., blinded analysis procedure). All samples were analyzed on the same assay for each analyte to eliminate inter-assay variance.

### Dietary habits

Participants were instructed to record the estimated quantities of all food and beverages consumed for at least 3 days per week (2 week-day and 1 day on the weekend), to offer a reliable estimate of food intake for the entire week. Total water intake was defined as the fluid volume of consumed beverages plus the water content of consumed foods.

A nutritional tracking application^1^ and the food composition tables of the National Institute of Statistics of Tunis (1978) were used to determine the distribution of macronutrients and the calories ingested. Each food item was individually entered into the application to provide data on total energy consumption, and the amounts of energy derived from proteins, fats, and carbohydrates for each period analyzed. The mean of nutrients consumed during the intervention was calculated using the method described by McCance and Widdowson (38).

Protein intake was supervised by a specialist to ensure that dietary protein needs were met. Subjects were instructed to consume dietary supplements on the training days containing 24 g of protein and 1 g of carbohydrate before sleeping (Iso100 Hydrolyzed Whey Protein Isolate; Dymatize Nutrition, Dallas, TX). There were no differences in the estimated nutrients consumed during the study between the experimental groups (Table 1).

**Table 1:** Estimated daily dietary intake (mean ± SD) recorded at T0, T1, T2, and T3 in FAST and FED groups (*N* = 37).

	Group	Phases				*p* values (ES)		
		T0	T1	T2	T3	Time	Group	Group × Time
Protein (% kcal)	FAST	18 ± 7	18 ± 9	18 ± 3	18 ± 8	0.07 (0.43)	0.07 (0.56)	0.1 (0.57)
	FED	18 ± 3	18 ± 7	18 ± 9	18 ± 7			
Carbohydrate (% kcal)	FAST	48 ± 2	47 ± 8	47 ± 3	47 ± 2	0.1 (0.35)	0.06 (0.54)	0.08 (0.45)
	FED	48 ± 3	46 ± 9	46 ± 9	46 ± 7			
Fat (% kcal)	FAST	36 ± 8	36 ± 5	36 ± 3	35 ± 7	0.09 (0.52)	0.1 (0.38)	0.08 (0.43)
	FED	36 ± 9	36 ± 3	37 ± 5	36 ± 5			
Energy (kcal/day)	FAST	3,456 ± 265	3,449 ± 270	3,435 ± 291	3,456 ± 275	0.2 (0.43)	0.08 (0.55)	0.08 (0.46)
	FED	3,481 ± 268	3,431 ± 237	3,471 ± 218	3,472 ± 232			
Total water intake (L/day)	FAST	4.3 ± 0.8	4.2 ± 0.4	4.3 ± 0.5	4.2 ± 0.7	0.06 (0.57)	0.08 (0.35)	0.1 (0.36)
	FED	4.2 ± 0.9	4.1 ± 0.7	4.3 ± 0.2	4.1 ± 0.9			

### Statistical analyses

Data were analyzed using SPSS software (v. 16.0, SPSS Inc., Chicago, IL, United States) and were expressed as means ± standard deviations (SD). The normality of data distribution was confirmed using the Shapiro–Wilk test. The effects of time of training during RF were evaluated using a 2 (groups: FAST, FED) × 4 (time: T0, T1, T2, T3) mixed model ANOVA. Additionally, hormonal data were assessed using a 2 (groups: FAST, FED) × 3 (time: BF, AF, AF-30 min) mixed model ANOVA. Where significant group-by-time interactions occurred, *post hoc* tests were computed using the Bonferroni adjustments to identify group-specific changes over time.

Effect sizes (ES) were calculated using ANOVA output by converting partial eta-squared η_p_^2^ to Cohen’s *d* values. In addition, within-group ES were computed using the following equation: ES = (mean post—mean pre)/SD. ES were considered trivial (<0.2), small (0.2–0.6), moderate (0.6–1.2), large (1.2–2, 0), and very large (2.0–4.0) (39). The level of statistical significance was set at *p* < 0.05. Means, standard deviations, and 95% confidence intervals (CI) were presented for all data.

## Results

Three subjects (one from FED and two from FAST) were not included in the final analyses due to low compliance (<90%). The remaining subjects (*N* = 37) met the 100% adherence requirement for continued participation in the study. There were no between-group differences for any anthropometric measures, strength parameters, and diurnal variation of hormonal secretions at baseline between the two groups. ES magnitudes ranged from ignored, small to moderate for all measurements.

### Anthropometric tests

Means, standard deviations, and 95% CI of anthropometric parameters and body composition characteristics are presented in Table 2 for the study sample. No significant group × time interactions were found for all anthropometric measurements. However, a significant main time effect was reported for both FAST and FED for body mass (*p* = 0.001; ES = 0.37), BMI (*p* = 0.001; ES = 0.32), and body fat (*p* = 0.001; ES = 0.41) during Ramadan, with no significant main time effect for lean body mass (*p* = 0.57; ES = 0.42).

**Table 2:** Anthropometric and body composition characteristics (means ± SD) at T0, T1, T2 and T3 in FAST and FED groups (N=37).

Variables	Group	Phases				p values (ES)		
		**T0**	**T1**	**T2**	**T3**	**Time**	**Group**	**Group x Time**
Body mass (kg)	FAST	78.88±3.65	75.79±3.05*	74.01±3.14*#	77.10±4.68**$**	**0.001 (0.37)**	**0.06 (0.24)**	**0.79 (0.12)**
	FED	80.00±3.53	78.81±3.18*	76.08±2.99*	78.61±3.54**$**			
BMI (kg/m^2^)	FAST	24.16±1.24	23.66±1.29*	22.35±1.31*#	23.66±1.15**$**	**0.001 (0.32)**	**0.28 (0.29)**	**0.58 (0.20)**
	FED	24.69±1.48	23.94±1.13	23.26±1.28*	24.02±1.25**$**			
Body fat (%)	FAST	16.61±1.14	15.38±1.52*	13.84±1.45*#	14.90±1.29*$	**0.001 (0.41)**	**0.98 (0.12)**	**0.4 (0.17)**
	FED	16.34±0.53	15.38±1.52*	13.95±1.56*#	15.01±1.34*$			
LBM (kg)	FAST	66.29±2.40	65.15±3.00	64.64±3.09	65.31±3.54	**0.57 (0.42)**	**0.06 (0.21)**	**0.84 (0.17)**
	FED	67.02±2.08	66.61±2.61	66.56±2.72	67.22±3.03			

**BMI:** body mass index; **LBM:** lean body mass; *: p< 0.05 from the corresponding time point T0; **#:** p< 0.05 from the corresponding time point T1; **$:** p<0.05 from the corresponding time point T2.

### Maximal strength tests

Table 3 contains the primary findings with regards to maximal strength performance. There were significant group × time interactions for 1-RM_SQ_ (*p* = 0.001; ES = 0.43) and 1-RM_DL_ (*p* = 0.001; ES = 0.36). *Post-hoc* tests indicated significant increases for 1-RM_SQ_ (*p* = 0.03; ES = 0.13) and 1-RM_DL_ (*p* = 0.04; ES = 0.21) from T0-T2 among the FED group. No significant group × time effect was found for 1-RM_BP_ (*p* = 0.68; ES = 0.21). At T3, significant improvements were observed for 1-RM_SQ_ (*p* = 0.04; ES = 0.24), 1-RM_BP_ (*p* = 0.04; ES = 0.20), and 1-RM_DL_ (*p* = 0.02; ES = 0.18) in the FED group and for 1-RM_SQ_ (*p* = 0.02; ES = 0.18), 1-RM_BP_ (*p* = 0.02; ES = 0.23), and 1-RM_DL_ (*p* = 0.04; ES = 0.19) in the FAST group in comparison with T0.

**Table 3:** One-repetition maximum (1-RM) changes (means ± SDs) at T0, T1, T2 and T3 in FAST and FED groups (N=37).

Variables	Group	Phases				p values (ES)		
		**T0**	**T1**	**T2**	**T3**	**Time**	**Group**	**Group x Time**
1-RM_SQ_ (kg)	FAST	169.41±14.67	170.29±13.13	167.24±12.60	172.53±11.79*#	**0.001 (0.35)**	**0.39 (0.12)**	**0.001 (0.43)**
	FED	170.47±11.61	173.21±11.75	175.56±11.35*	178.00±12.35*#			
1-RM_BP_ (kg)	FAST	106.47±10.21	110.94±11.70	110.29±10.87	120.12±11.27*	**0.001 (0.48)**	**0.66 (0.13)**	**0.68 (0.21)**
	FED	105.29±10.97	108.06±10.88	110.59±11.14	117.06±10.76*			
1-RM_DL_ (%)	FAST	187.06±8.89	187.06±9.83	187.65±9.86	188.65±8.17*#	**0.001 (0.43)**	**0.04 (0.21)**	**0.001 (0.36)**
	FED	187.94±7.96	191.88±7.88	197.24±8.22*	199.59±8.67*#			

**1-RM:** one-repetition maximum; **SQ:** squat; **BP:** bench press; **DL:** deadlift; **QC:** quadriceps; **BB:** biceps brachii; *: p< 0.05 from the corresponding time point T0; **#:** p< 0.05 from the corresponding time point T1; **$:** p<0.05 from the corresponding time point T2.

### Sleep quality

Means, standard deviations, and 95% confidence intervals (CI) of the Pittsburgh Sleep Quality Index at T0, T1, T2, and T3 among FAST or FED groups are reported in Table 4. No significant group × time interaction was found for the total score of the PSQI (*p* = 0.07; ES = 0.43). There was a main effect of time for the total score of PSQI. A higher global PSQI score was observed RF in both groups at T1 (*p* = 0.02; ES = 0.36 for FAST and *p* = 0.04; ES = 0.26 for FED) and T2 (*p* = 0.001; ES = 0.46 for FAST and *p* = 0.02; ES = 0.35 for FED) compared to T0. Total PSQI scores were lower 21 days after RF (T3) compared to T1 (*p* = 0.01; ES = 0.32 for FED and *p* = 0.01; ES = 0.27 for FAST) and T2 (*p* = 0.001; ES = 0.16 for FED and *p* = 0.03; ES = 0.18 for FAST).

**Table 4:** Sleep quality (mean ± SD) recorded at T0, T1, T2, and T3 in the FAST and FED groups (*N* = 37).

	Group	Phases				*p* values (ES)		
		T0	T1	T2	T3	Time	Group	Group × Time
Sleep quality	FAST	1.2 ± 0.6	2.3 ± 0.8*	2.3 ± 0.7*	1.3 ± 0.7**#$**	**0.006 (0.58)**	**0.08 (0.36)**	**0.09 (0.42)**
	FED	1.1 ± 0.7	2.4 ± 0.4*	2.4 ± 0.5*	1.2 ± 0.6**#$**			
Sleep latency	FAST	0.7 ± 0.4	0.7 ± 0.2	0.8 ± 0.4	0.7 ± 0.4	**0.07 (0.37)**	**0.1 (0.47)**	**0.1 (0.46)**
	FED	0.8 ± 0.1	0.7 ± 0.4	0.7 ± 0.2	0.8 ± 0.2			
Sleep duration	FAST	1.1 ± 0.5	1.3 ± 0.2*	1.3 ± 0.9*#	1.2 ± 0.5**#$**	**0.001 (0.45)**	**0.2 (0.34)**	**0.07 (0.65)**
	FED	1.1 ± 0.7	1.2 ± 0.3*	1.3 ± 0.7*#	1.1 ± 0.2**#$**			
Sleep efficiency	FAST	0.8 ± 0.2	0.8 ± 0.6*	0.8 ± 0.5*	0.7 ± 0.4**#$**	**0.004 (0.55)**	**0.07 (0.24)**	**0.08 (0.67)**
	FED	0.7 ± 0.5	0.8 ± 0.7*	0.8 ± 0.8*	0.7 ± 0.2**#$**			
Sleep disturbance	FAST	0.7 ± 0.5	0.7 ± 0.4	0.7 ± 0.5	0.6 ± 0.8	**0.08 (0.35)**	**0.08 (0.62)**	**0.1 (0.55)**
	FED	0.6 ± 0.9	0.7 ± 0.3	0.6 ± 0.7	0.6 ± 0.7			
Daytime dysfunction	FAST	0.4 ± 0.7	0.4 ± 0.4	0.4 ± 0.8	0.5 ± 0.1	**0.07 (0.45)**	**0.07 (0.38)**	**0.08 (0.56)**
	FED	0.5 ± 0.3	0.4 ± 0.7	0.5 ± 0.8	0.4 ± 0.3			
Total score of PSQI	FAST	3.7 ± 2.1	7.1 ± 2.3*	7.2 ± 2.1*	3.8 ± 2.2**#$**	**0.002 (0.42)**	**0.07 (0.56)**	**0.07 (0.43)**
	FED	3.7 ± 2.3	7.3 ± 2.1*	7.2 ± 2.6*	4.1 ± 1.1**#$**			

PSQI, Pittsburgh Sleep Quality Index.*p < 0.05 from the corresponding time point T0; ^**#**^*p* < 0.05 from the corresponding time point T1; ^$^*p* < 0.05 from the corresponding time point T2.

### Blood samples

**Figure 3:**
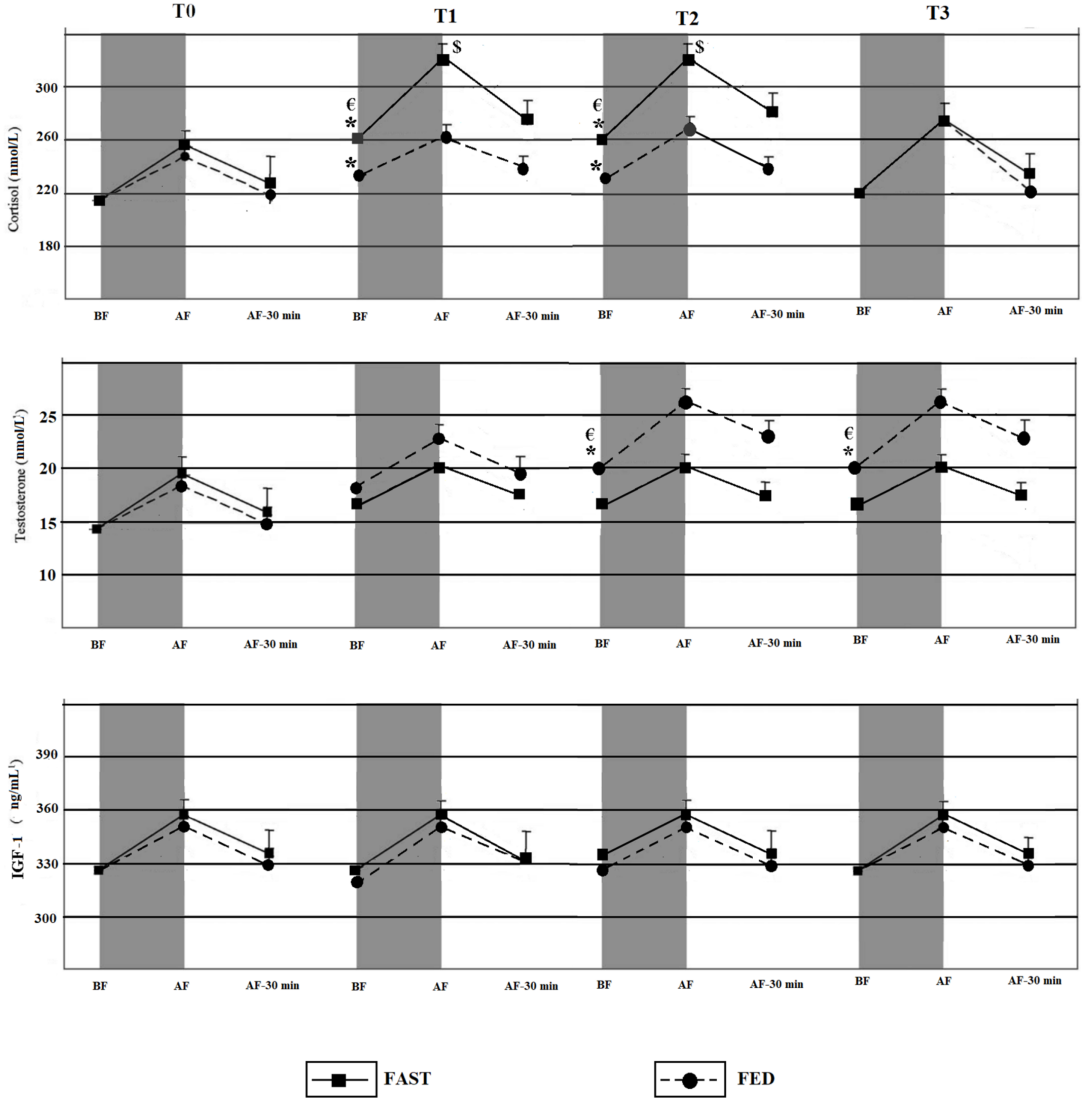
Acute and chronic effects of training during RF among FED and FAST groups. BF, Before exercise; AF, Immediately after exercise; AF-30 min, 30 min after exercise. *Significant time effect compared to T0 (*p* < 0.05); ^€^Significant group × time chronic effect of training (*p* < 0.05); ^$^significant group × time acute effect of exercise (*p* < 0.05).

Means, standard deviations, and 95% confidence intervals (CI) of the acute and chronic effects of exercise (FED, FAST) during RF are illustrated in Figure 3.

Significant group × time interactions were identified for the chronic RT effects on measures of cortisol (*p* = 0.03; ES = 0.27). *Post-hoc* tests indicated significant increases in cortisol levels in the FAST group at T1 and T2 compared to T0 (*p* = 0.05; ES = 0.41 and *p* = 0.03; ES = 0.34). Significant increases in cortisol levels also occurred in the FED group at T1 (*p* = 0.05; ES = 0.29) and T2 (*p* = 0.04; ES = 0.25) compared to T0. However, the increases were significantly lower compared to the FAST group. Cortisol levels decreased after the Ramadan, and no significant differences were found for cortisol level at T3 compared with T0 among both groups (*p* > 0.05).

Significant group × time interactions occurred with regards to the chronic RT effects for testosterone levels (*p* = 0.01; ES = 0.32). *Post-hoc* tests indicated significant increases of testosterone among FED at T2 and T3 compared to T0 (*p* = 0.04; ES = 0.31 and *p* = 0.03; ES = 0.42). Additionally, testosterone levels remained significantly elevated after the end of RF at T3 vs. T0 (*p* = 0.05; ES = 0.28) and T3 vs. T1 (*p* = 0.03; ES = 0.25).

A significant group × time interaction occurred for cortisol levels (*p* = 0.04; ES = 0.34) for the acute effects of exercise. The level of cortisol immediately after RT was higher at T1 (*p* = 0.03; ES = 0.39) and T2 (*p* < 0.05; ES = 0.22) compared with T0 among the FAST group. However, there were no significant differences in the cortisol levels between T0 and T3 (i.e., for the acute effects of exercise) in the FAST group, immediately after RT.

No significant group × time interactions were detected for IGF-1 (*p* = 0.07; ES = 0.27).

## Discussion

This study was conducted with healthy young and RT experienced males. In this study, we aimed to evaluate the effects of RT applied during RF on muscular strength, sleep quality, and selected blood hormonal concentrations (chronic and acute effects) in a FED state vs. in a FAST state. To the best of our knowledge, this is the first study that investigates the importance of choosing the adequate day time RT training for a better muscle performance, taking into consideration the effects on sleep quality and hormonal secretions.

The key findings of our study include: (1) significant improvements for 1-RM_DL_ and 1-RM_SQ_ only in FED during RF (T1), however, while after the end of RF (T2) 1-RM_DL_, 1-RM_SQ_, and 1-RM_BP_ improved in both groups (2) significant chronic effects on hormonal concentrations was detected in both groups, cortisol level increased among FAST at the second day of RF (T1) and lasted till its end (T2), while testosterone increased in FED at the last week of the RF (T1) and remained to increase even after the end of RF (T3); (3) significant increases in cortisol levels were reported only among FAST, increased cortisol levels immediately after RT sessions during RF (T1) were higher than before the start of RF, while they returned to the normal level after the end of RF (T2). No significant between group differences were observed for sleep quality during the experiment.

The observed significant deadlift and squat improvements were found in the FED group only and might be due to adequate pre-exercise energy and fluid intake levels. The RT sessions of FED occurred at 21:00 h under favorable conditions as the athletes were able to refuel and rehydrate for ∼90 min before the exercise sessions, while the FAST group trained after at least 13 h of fasting. Previous studies reported that the athletes’ pre-exercise blood glucose concentration levels and fluid balance were within the normal range during the evenings, while training at 18:00 h was the least favorable time of day for training as the athletes abstained from food and fluid for more than 10 h (40).

The effects of RT applied during RF on measures of muscle strength were also reported by others who obtained similar results (41, 42). Authors from previous studies indicated that the optimal time of day to perform high-intensity exercises during RF is in the evening, after breaking the fast. Training-induced adaptations might be diminished during the fasting state of RF (25, 40).

Hormonal changes were also reported in our study, and showed significant increases in cortisol levels in FAST likely due to a chronic effect of training during fasting. Increases in cortisol levels were also higher after breaking fast (FED) compared to before RF levels, but remained within ranges. The modification of the time difference between the food and water intakes and training sessions could underly increases in cortisol levels in FAST compared with pre-RF levels and to the FED state group. The higher level of cortisol concentration during fasting is a response to a catabolic process to restore glucose needs by stimulating gluconeogenesis, proteolytic activity and increasing skeletal protein degradation after 12 h of fasting (44). Increases in cortisol levels during RF were also reported previously (45–47). However, some studies observed decreases in cortisol levels in judo athletes after a higher training load during RF (12). One reason for this discrepancy could be the timing of blood sampling collection, which occurred between 8:00 h and 10:00 h in the later study (12), and in the afternoon as in our study.

The cortisol levels immediately after a single bout of RT in FAST were also higher compared to pre-RF levels. Differences in the magnitude of acute RT-induced cortisol responses may be due to the effects of a new physiological stress (fasting) that could lead to increased catabolic processes to mobilize fatty acids from fat reserves, and activation of protein breakdown from muscle tissue to raise blood glucose concentrations during food restriction during exercise in the FAST state (48). Similar study results were reported previously. The reported increases in cortisol levels after exercise in a fasting state (caloric restriction) were greater than in fed state (30, 49).

Significant increases of testosterone levels were only found in the FED state group during RF, which may favor physiological responses to modulate the balance between hormone-mediated anabolic and catabolic activities after the slight increase of cortisol concentrations during RF. Thus, the observed increase in muscle strength in FED can be explained by favoring anabolic functions of testosterone in skeletal muscle and neuronal tissue (50, 51). Similar findings were reported by Mira et al. (52) who observed significant increases in testosterone during RF in the evening hours and decreases in the morning. These findings were explained by the effects of RF on changes in the circadian rhythm and metabolic regulation during RF (52). The effects of fasting and training were also investigated in other studies, where testosterone levels were unchanged when measured before breaking the fast during RF (17, 53). To the best of our knowledge, no study investigated the effects of training times on testosterone secretion during RF.

Additionally, there were continued increases in testosterone levels only in the FED group even after the end of Ramadan (T3) in comparison with T0 and T1. Researchers from previous studies reported elevations in testosterone levels after long-term resistance training (8 weeks of training) in a non-fasting state while there were no increases in the FAST group (T3) undergoing only 3 weeks of training in a fed state (45, 46).

There were non-significant changes in IGF-1 levels in the two study groups. IGF-1 levels can be affected by food restriction and dehydration during RF or by changes in physical activity levels (54, 55). This was not the case in our study where there were no differences in total energy and water intake, or in total training volume between the two study groups. However, this non-significant change (tendency to decrease) may be due to increased cortisol levels which can decrease the skeletal IGF-I synthesis (44).

Sleep is an essential component of improving performance by promoting post-training recovery (56). Previous studies indicated that training at night causes sleep disturbances due to high pre-sleep arousal levels, especially if it occurs during 3 h before going to bed (57). Researchers from other studies reported that RF affects the circadian rhythm and produces modifications in the sleep quality with significant reductions in total sleep time (58, 59).

Our results showed a non-significant group × time effect for the factor day time training on sleep quality. However, significant main time effects were found for sleep quality during RF fasting. In fact, the Ramadan month affords substantial lifestyle adjustments and traditions involving longer nightly prayers, sleep disturbances due to the timing of *suhur* meal with poor sleep experiences.

### Study limitations

This study was conducted during the COVID partial lock down period, and training sessions for the FED group occurred between 20:00 h and 22:00 h before gym closing times (as mandated by the Tunisian Government), making it important to study late-night RT and its effects on sleep quality and hormonal secretion.

Additionally, it could be useful to add a third day-time point to investigate the effects of RT after a short time after starting fasting. In fact, a morning training in a FAST state, after a short time of taking *suhur* meal could provide important insights.

### Practical applications

Our study examined the effects of RT during RF on body composition, strength performance, and hormonal secretion. The results of our study suggest that RT during RF induces favorable changes in body composition (loss of body fat, maintaining lean tissue). Moreover, practicing RT after breaking fast (Fed state) improved muscle strength, probably due to a more favorable anabolic and hormonal status compared with the FAST. Thus, the results of this study can guide coaching strategies for strength and conditioning specialists. Choosing the appropriate time of day to practice RT training during RF is an important consideration for recreational weightlifters, and also for athletes from other (strength-dominated) sports to avoid physiological and psychological challenges that occur during this month of daily fasting.

## Conclusion

The aim of this study was to investigate the appropriate time of day for recreational athletes who continue to train during RF to maintain and improve muscle performance in order to minimize the effects of meal timing changes on muscle performance. Our study demonstrated that RT applied after breaking fast in a FED state improved energy intake and testosterone secretion, thus enhancing muscle strength. Additionally, practicing RT in a FAST state had no adverse effects on measures of muscle strength when maintaining the same training volume and energy intake.

## Data Availability

The raw data supporting the conclusions of this article will be made available by the authors, without undue reservation.

## Footnotes

^1^http://www.myfitnesspal.com
